# Evaluation of plasma cytokines in patients with cocaine use disorders in abstinence identifies transforming growth factor alpha (TGFα) as a potential biomarker of consumption and dual diagnosis

**DOI:** 10.7717/peerj.3926

**Published:** 2017-10-12

**Authors:** Rosa Maza-Quiroga, Nuria García-Marchena, Pablo Romero-Sanchiz, Vicente Barrios, María Pedraz, Antonia Serrano, Raquel Nogueira-Arjona, Juan Jesus Ruiz, Maribel Soria, Rafael Campos, Julie Ann Chowen, Jesus Argente, Marta Torrens, Meritxell López-Gallardo, Eva María Marco, Fernando Rodríguez de Fonseca, Francisco Javier Pavón, Pedro Araos

**Affiliations:** 1 Hospital Regional Universitario de Málaga, Unidad de Gestión Clínica de Salud Mental, Instituto de Investigación Biomédica de Málaga (IBIMA), Málaga, Spain; 2 Department of Endocrinology, Hospital Universitario Niño Jesús, Madrid, Spain; 3 Diputación de Málaga, Centro Provincial de Drogodependencias, Málaga, Spain; 4 Institut de Neuropsiquiatria i Addiccions (INAD) del Parc de Salut Mar, Barcelona, Spain; 5 Department of Physiology Faculty of Medicine, Universidad Complutense de Madrid, Madrid, Spain; 6 Department of Physiology II Faculty of Biology, Universidad Complutense de Madrid, Madrid, Spain

**Keywords:** Cocaine use disorders, Cytokines, Dual diagnosis

## Abstract

**Background:**

Cocaine use disorder (CUD) is a complex health condition, especially when it is accompanied by comorbid psychiatric disorders (dual diagnosis). Dual diagnosis is associated with difficulties in the stratification and treatment of patients. One of the major challenges in clinical practice of addiction psychiatry is the lack of objective biological markers that indicate the degree of consumption, severity of addiction, level of toxicity and response to treatment in patients with CUD. These potential biomarkers would be fundamental players in the diagnosis, stratification, prognosis and therapeutic orientation in addiction. Due to growing evidence of the involvement of the immune system in addiction and psychiatric disorders, we tested the hypothesis that patients with CUD in abstinence might have altered circulating levels of signaling proteins related to systemic inflammation.

**Methods:**

The study was designed as a cross-sectional study of CUD treatment-seeking patients. These patients were recruited from outpatient programs in the province of Malaga (Spain). The study was performed with a total of 160 white Caucasian subjects, who were divided into the following groups: patients diagnosed with CUD in abstinence (*N* = 79, cocaine group) and matched control subjects (*N* = 81, control group). Participants were clinically evaluated with the diagnostic interview PRISM according to the DSM-IV-TR, and blood samples were collected for the determination of chemokine C-C motif ligand 11 (CCL11, eotaxin-1), interferon gamma (IFNγ), interleukin-4 (IL-4), interleukin-8 (IL-8), interleukin-17α (IL-17α), macrophage inflammatory protein 1α (MIP-1α) and transforming growth factor α (TGFα) levels in the plasma. Clinical and biochemical data were analyzed in order to find relationships between variables.

**Results:**

While 57% of patients with CUD were diagnosed with dual diagnosis, approximately 73% of patients had other substance use disorders. Cocaine patients displayed greater cocaine symptom severity when they were diagnosed with psychiatric comorbidity. Regarding inflammatory factors, we observed significantly lower plasma levels of IL-17α (*p* < 0.001), MIP-1α (*p* < 0.001) and TGFα (*p* < 0.05) in the cocaine group compared with the levels in the control group. Finally, there was a significant primary effect of dual diagnosis on the plasma concentrations of TGFα (*p* < 0.05) in the cocaine group, and these levels were lower in patients with dual diagnoses

**Discussion:**

IL-17α, MIP-1α and TGFα levels are different between the cocaine and control groups, and TGFα levels facilitate the identification of patients with dual diagnosis. Because TGFα reduction is associated with enhanced responses to cocaine in preclinical models, we propose TGFα as a potential biomarker of complex CUD in humans.

## Introduction

Cocaine use disorder (CUD) constitutes a highly complex health problem involving not only biological changes in the brain but also a variety of social and environmental aspects (Tomasi et al., 2015). The chronic use of cocaine is often accompanied by medical issues, including circulatory diseases and comorbid psychiatric conditions (González-Saiz et al., 2014; Verdejo-Garcia et al., 2015; Chibungu et al., 2016). Dual diagnosis or co-occurring disorders describes the presence of both a mental health and a substance use disorder, and the dual nature of this condition complicates the diagnosis and therapeutic outcomes in CUD because many of the patients do not respond to classical pharmacological approaches (Torrens, Gilchrist & Domingo-Salvany, the PsyCoBarcelona Group, 2011; Álvarez et al., 2013). Moreover, the quality of life of these patients is severely impaired (Chahua et al., 2015).

To improve the therapeutic process in addiction, one of the challenges is to identify biological markers that might aid in objectively determining the degree of consumption, severity of addiction, level of toxicity and response to treatment in patients with CUD (Pavón et al., 2013; Araos et al., 2015). These potential biomarkers would be fundamental players in the diagnosis, stratification, prognosis and therapeutic orientation in addictions. Several attempts have been made to identify these possible biomarkers by exploring accessible signaling compartments (i.e., blood) or functional neuropsychological tests. Among these attempts, recent studies have identified circulating factors involved in immune function as potential biomarkers in CUD and other substance use disorders (Araos et al., 2015; Moreira et al., 2016; García-Marchena et al., 2017a). These and other studies have demonstrated that abused drugs interact with the immune system and alter signaling and gene expression involved in the immune response, which these effects contribute to various aspects of addiction (Cui, Shurtleff & Harris, 2014).

More specifically, cocaine abuse results in increased pro-inflammatory signaling throughout the brain (Cearley et al., 2011; Coller & Hutchinson, 2012); however, the mechanism is unknown. These alterations are also found in some plasma circulating chemokines and cytokines (Araos et al., 2015), indicating that alterations in pro- and anti-inflammatory factors may serve as potential biomarkers of an inflammatory state in the central nervous system (CNS). In fact, this systemic inflammation has been recently proposed to be a mechanism that facilitates dopamine signaling in the brain, which contributes to the addiction cycle (Petrulli et al., 2017).

The fact that the immune system modifies brain functions related to addiction and the concurrent participation of reward modulatory systems in psychiatric disorders open the possibility of establishing a link between inflammation, neuropsychiatric diseases and addictive disorders (Stertz, Magalhaes & Kapczinski, 2013). Comorbid mental health and substance use disorders are usually present in more than 50% of cocaine- or alcohol-addicted patients (Vergara-Moragues et al., 2012; García-Marchena et al., 2017b). Convergence of neuroinflammation, stress and addiction in neural substrates of mental diseases could help us to understand this complex association. Stressful experiences may model the immune system, modifying the release and signaling of cytokines involved in inflammation (Slavich, 2016). In addition, several studies indicate that the inflammatory processes could actively influence the CNS and that this would generate changes in the behavior of the subjects (Irwin & Miller, 2007). As an example, neuroinflammation and alterations in neurogenesis are clear substrates of mood disorders (Goldstein et al., 2009), and both processes are clearly affected by abused drugs (Castilla-Ortega et al., 2016; Neupane, 2016).

This research is the continuation of a previous study in patients with CUD (Araos et al., 2015); in the present study, we also analyzed the inflammatory signaling factors in the blood of patients addicted to cocaine to explore the relationships between the expression of circulating agents related to neuroinflammation and neurogenesis (cytokines, chemokines and growth factors) and variables associated with CUD and dual diagnosis. The selected molecules in these two studies were chosen according to an exhaustive bibliographic search.

The inflammatory signals examined in the current study were chemokine C-C motif ligand 11 (CCL11, eotaxin-1), *interferon gamma* (IFNγ), interleukin-4 (IL-4), interleukin-8 (IL-8), interleukin-17α (IL-17α), macrophage inflammatory protein 1α (MIP-1α) and transforming growth factor α (TGFα).

The main goal of the present study was to evaluate the plasma concentrations of cytokines, chemokines and growth factor in a cohort of CUD patients in abstinence compared to population controls. Variables related to CUD, such as the duration of problematic cocaine use, abstinence length, cocaine addiction severity and dual diagnosis, were also examined in relation to these molecules.

## Materials and Methods

### Study design and recruitment

A cross-sectional study was conducted on patients diagnosed with CUD seeking treatment for cocaine use compared with control subjects. The cocaine patients were recruited from outpatient treatment programs in the province of Malaga (Spain) for a period of 24 months (August 2014 to August 2016). A total of 160 white Caucasian subjects were recruited and divided into the cocaine group and the control group. To be eligible for the study, participants had to be ≥18 years to 65 years of age. Exclusion criteria included a personal history of the following: (1) chronic diseases (e.g., cardiovascular, respiratory, renal, hepatic, neurological or endocrinological diseases); (2) cancer; (3) infectious diseases; (4) incapacitating cognitive alterations; and (5) pregnancy for women.

### Cocaine group and other dual diagnoses

Seventy-nine abstinent patients were diagnosed with CUD (cocaine abuse and/or dependence). The diagnosis of CUD and other psychiatric disorders were performed with a psychiatric interview (‘Diagnostic and Statistical Manual of Mental Disorders-4th Edition-Text Revision’, DSM-IV-TR), while the abstinence from abused drugs was controlled weekly by urine analysis in the outpatient treatment centers for cocaine addiction. Urine analysis for cocaine, amphetamine, opiates, barbiturates, phencyclidine and cannabis was performed using a V-Twin Drug Testing System (Siemens AG, Erlangen, Germany). These data were used to select patients with CUD who abstained from abused drugs during the last 2 weeks (at least). Subsequently, plasma analyses were conducted to verify cocaine abstinence.

### Control group without dual diagnosis

The control group was recruited from a multidisciplinary staff working at the *Hospital Regional Universitario de Málaga* (Málaga, Spain). Eighty-one healthy and unmedicated participants were matched to the cocaine group for age, sex and body mass index (BMI) in order to provide reference values of circulating inflammatory factors. Subjects with substance use disorders and past or current Axis I and Axis II disorders (DSM-IV-TR) and neurologic disorders were excluded from the control group.

### Ethics statement

Written informed consent was obtained from each participant after a complete description of the study and after questions or issues were discussed. The study and protocols for recruitment were approved by the Ethics Committee of the *CEI Provincial de Málaga* in accordance with the ‘Ethical Principles for Medical Research Involving Human Subjects’ that was adopted in the Declaration of Helsinki by the World Medical Association (64th WMA General Assembly, Fortaleza, Brazil, October 2013). The data were protected by the Recommendation *No*. *R* (*97*) *5* of the Committee of Ministers to Member States on the Protection of Medical Data (1997) and the Spanish data protection act (*Ley Orgánica 15/1999 de Protección de Datos, LOPD)*.

### Psychiatric evaluation

All patients with CUD were evaluated according to the DSM-IV-TR criteria using the Spanish version of the ‘Psychiatric Research Interview for Substance and Mental Disorders’ (PRISM 6.0) (Hasin et al., 1996; Torrens et al., 2004). Control subjects were initially evaluated by PRISM to detect substance use disorders and by the Spanish version of ‘Dual Diagnosis Screening Instrument’ (DDSI) to detect psychiatric disorders (DSM-IV-TR Mestre-Pintó et al., 2014). All interviews were conducted by psychologists who had received training certificates for these instruments.

#### PRISM

The PRISM diagnostic interview was used to assess psychopathological and substance use disorders. PRISM is a semi-structured clinical interview designed to solve the problems of diagnosis in people with consumption of substances and/or alcohol. This interview presents good test-retest reliability, validity and inter-examiner reliability (kappa coefficient oscillates between 0.66 and 1.00) (Morgello et al., 2006). The first module contains questions related to the history of consumption, ranging from the time of the diagnosis of abuse and/or dependence. In addition, PRISM evaluates 20 Axis I disorders and 2 Axis II disorders (borderline and antisocial personality disorders) according to DSM-IV-TR. The diagnoses are performed using two time-frames: *current* (criteria were met within the past year) and *past* (criteria were met before the previous 12 months). Consequently, the estimated prevalence of a *lifetime* diagnosis would include both current and past diagnoses.

In addition, PRISM allows differentiation between primary mental disorders (or independent disorders) and disorders induced by substances in combination with the expected symptoms of the effect of intoxication and abstinence. The criteria established by PRISM for a substance-induced disorder is that the disorder must occur in the context of a pathological consumption of the substance in any of these 2 situations: (a) chronic intoxication (consumption for four or more days a week for a month) and (b) binge (consumption for a period of three continuous days) (Hasin et al., 1996; Torrens et al., 2004).

The cocaine severity symptom was used to determine cocaine trait severity combining the seven dependence criteria and the four abuse criteria (DSM-IV-TR) (Pavón et al., 2013; Araos et al., 2015; Pedraz et al., 2015).

### Collection of plasma samples for analysis

Blood samples were extracted in the morning (08:00–10:00 AM) after fasting for 8–12 h and before the psychiatric interview. Venous blood samples were extracted into 10-mL K2 EDTA tubes (BD, Franklin Lakes, NJ, USA) and centrifuged at 2,200 g for 15 min (4 °C) to obtain plasma. Plasma samples were individually assayed by three commercial tests for detecting infectious diseases: HIV, hepatitis B and hepatitis C. Plasma analyses for cocaine metabolite (Benzoylecgonine Specific Direct ELISA Kit purchased from Immunalysis Co., Pomona, CA, USA) were also performed to confirm cocaine abstinence. Plasma samples were stored at −80 °C.

### Multiplex immunoassay analysis

For plasma determination, inflammatory proteins were chosen considering those inflammatory factors that were not determined previously in abstinent cocaine patients (see details in Araos et al., 2015). A Bio-Plex Suspension Array System 200 (Bio-Rad Laboratories, Hercules, CA, USA) and ProcartaPlex Immunoassay Kit with magnetic beads and an appropriate Plasma Standard Diluent Kit (eBioscience, Affymetrix, Santa Clara, CA, USA) were used to quantify protein levels in the plasma. This method of analysis is based on the Luminex technology, and a human cytokine 7-plex panel (Mix&Match Panel) was used to simultaneously detect the following analytes: chemokine C-C motif ligand 11 (CCL11, eotaxin-1), interferon gamma (IFNγ), interleukin-4 (IL-4), interleukin-8 (IL-8), interleukin-17α (IL-17α), macrophage inflammatory protein 1α (MIP-1α) and transforming growth factor α (TGFα).

The measurements of these analytes in plasma were performed following the manufacturer’s instructions.

### Statistical analysis

**Figure 1:**
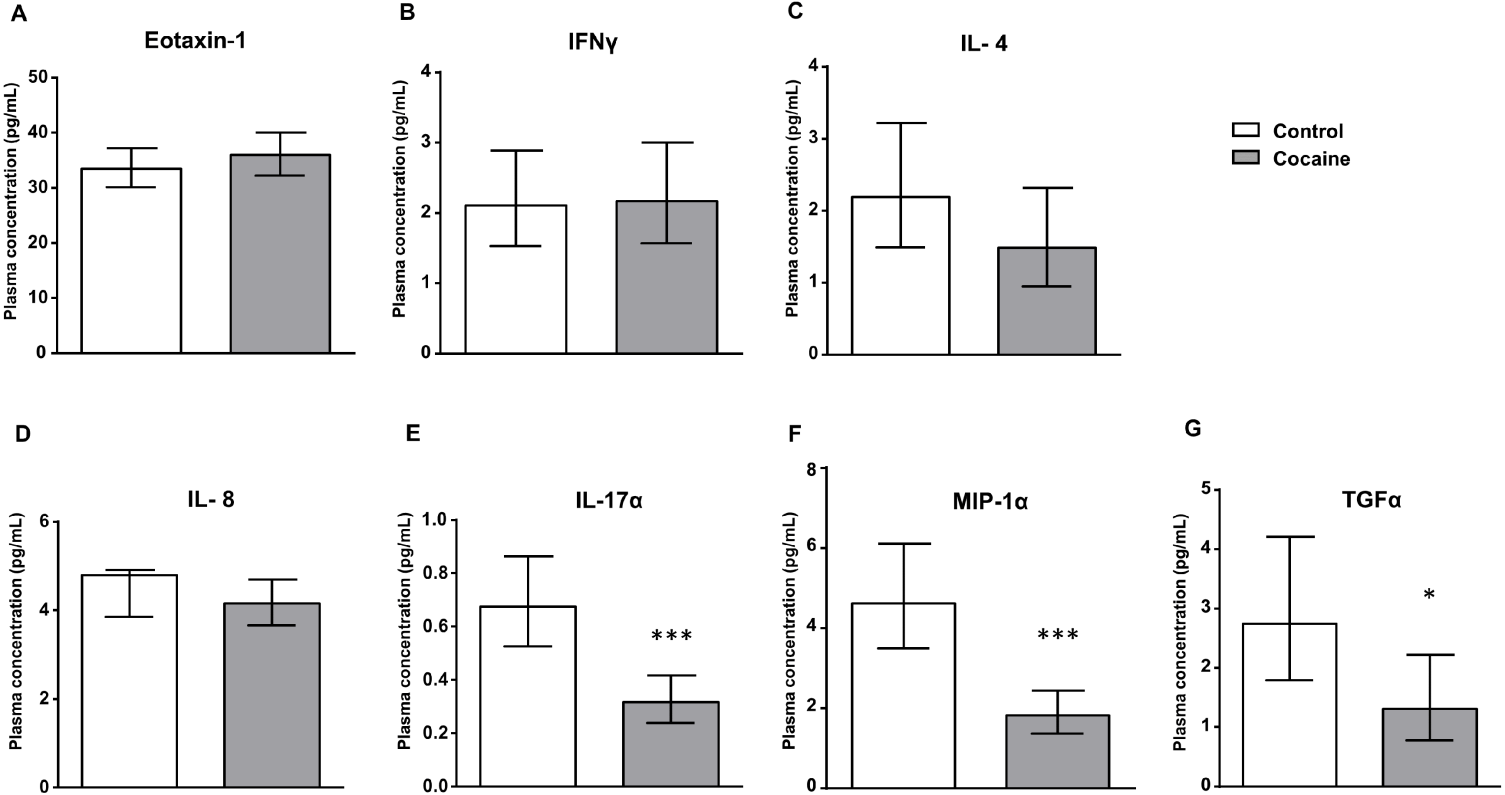
Cocaine vs. control group. Plasma levels of eotaxin-1 (CCL11), IFNγ, IL-4, IL-8, IL-17α, MIP-1α and TGFα in abstinent patients with CUD (cocaine group) and control subjects (control group). Bars are estimated marginal means and 95% CI (pg/mL). Data were analyzed by ANCOVA and (*) *p* < 0.05 and (***) *p* < 0.001 denote significant main effect of ‘cocaine use’, respectively.

**Figure 2:**
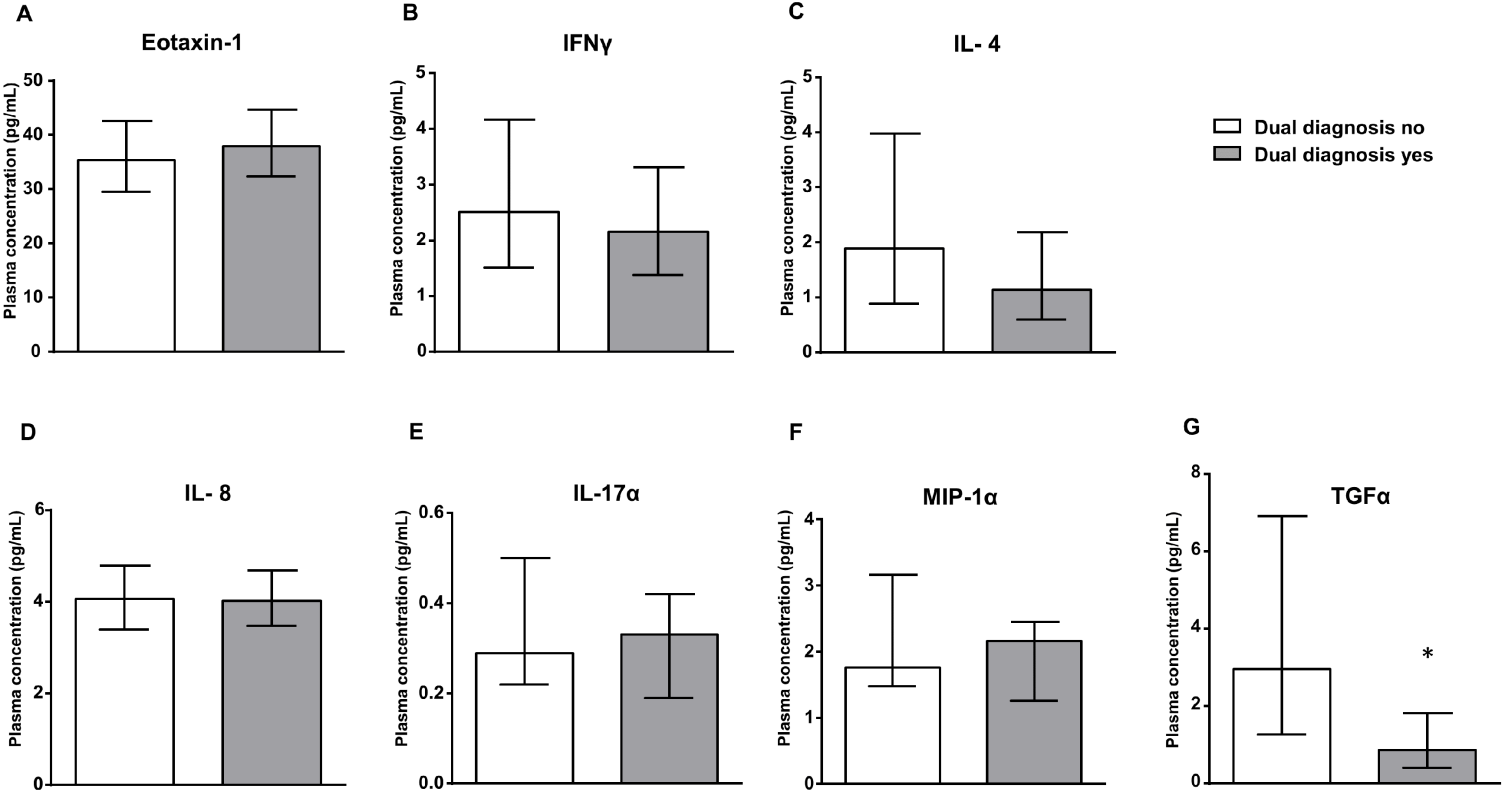
Dual diagnosis yes vs. dual diagnosis no. Plasma levels of eotaxin-1 (CCL11), IFN*γ*, IL-4, IL-8, IL-17*α*, MIP-1*α* and TGF*α* in the cocaine group according to dual diagnosis. Bars are estimated marginal means and 95% CI (pg/mL). Data were analyzed by ANCOVA and (*) *p* < 0.05 denotes significant main effect of ‘dual diagnosis’.

All clinical data in Tables 1 and 2 are expressed as the number and percentage of subjects (N (%)) or the mean and SD (mean (SD)). The significance of differences in categorical variables was determined using Fisher’s exact test (Chi-square test). The significance of differences in normal continuous variables or non-normal continuous variables was determined using Student’s *t*-test and Mann–Whitney *U* test, respectively. Statistical analysis of protein levels was performed using multiple analysis of covariance (ANCOVA) to indicate the relative effect of explanatory variables and their interactions on the protein expression in the plasma, controlling for additional covariates. Log (10) transformation was used to ensure statistical assumptions for positive skewed distributions. Estimated marginal means (95% confidence intervals [95% CI]) of protein levels were expressed after back-transformation as shown in Table 3 and Figs. 1 and 2.

**Table 1:** Baseline socio-demographics and psychiatric characteristics in cocaine and control group.

Variable		Total *N* = 160		
		Cocaine *N* = 79	Control *N* = 81	*p* value
Age (mean(SD))	*Years*	34.87 (7.18)	37.27 (10.97)	0.115^a^
BMI (mean(SD))	*Kg*/*m*^2^	25.88 (4.20)	25.32 (3.65)	0.370^b^
Sex (N(%))	Women	16 (20.25)	16 (19.75)	1^c^
	Men	63 (79.75)	65 (80.25)	
Marital status (N(%))	Single	29 (36.71)	26 (47.3)	0.004^c^
	Married/Cohabiting	29 (36.71)	22 (40.0)	
	Divorced/separated	20 (25.32)	7 (12.7)	
	Widowed	1 (1.27)	0 (0.00)	
Education (N(%))	≤Primary/elementary	63 (79.75)	9 (11.11)	<**0.001**^c^
	≥Secondary	16 (20.25)	72 (88.89)	
Psychiatric treatment (N(%))	No	52 (65.82)	72 (88.89)	<**0.001**^c^
	Yes	27 (34.18)	9 (11.11)	
Dual diagnosis (N(%))	No	34 (43.04)	–	–
	Yes	45 (56.96)		
Other substance use disorders (N(%))	No	21 (26.58)	–	–
	Yes	58 (73.42)		
Length of abstinence (mean (SD))	*Days*	133.4 (114.53)	–	–
Cocaine symptom severity (mean (SD))	*(0–11)criteria*	8.04 (2.66)	–	–

**Notes.**

Abbreviations BMI body mass index

a *p*-value from Student’s-test.

b *p*-value from Wilcoxon-test.

c *p*-value from Chi-square-test.

**Table 2:** Baseline socio-demographic variables and psychiatric characteristics in substance use disorders and dual diagnosis.

Variable		Cocaine *N* = 79					
		Other substance use disorders	Other substance use disorders	*p* value	Dual diagnosis	Dual diagnosis	*p* value
		Yes	No		Yes	No	
Participants (N (%))		58 (73.42)	21 (26.58)	–	45 (56.96)	34 (43.04)	–
Age (mean (SD))	*Years*	34.72 (7.15)	35.29 (7.42)	0.726^b^	36.53 (6.65)	32.68 (7.18)	**0.025**^b^
BMI (mean (SD))	*kg*/*m*^2^	25.58 (3.41)	26.70 (5.89)	0.300^a^	26.58 (4.61)	24.95 (3.43)	0.089^a^
Sex (N (%))	*Women*	10 (17.24)	6 (28.58)	0.430^c^	11 (24.44)	5 (14.71)	0.433^c^
	*Men*	48 (82.76)	15 (71.43)		34 (75.56)	29 (85.29)	
Psychiatric treatment (N(%))	*No*	37 (63.79)	15 (71.43)	0.716^c^	26 (57.78)	26 (76.47)	0.135^c^
	*Yes*	21 (36.21)	6 (28.58)		19 (42.22)	8 (23.53)	
Age of cocaine initiation (mean (SD))	*Years*	26.48 (8.12)	28.90 (7.47)	0.236^a^	26.42 (7.86)	28.06 (8.16)	0.507^b^
Length of abstinence (mean (SD))	*Days*	139.50 (113.20)	116.30 (119.26)	0.287^b^	133.5 (102.68)	133.2 (130.19)	0.334^b^
Cocaine symptom severity (mean (SD))	*(0–11)criteria*	8.41 (2.56)	7.00 (2.70)	<**0.001**^b^	9.20 (1.65)	6.50 (2.97)	<**0.001**^b^
Dual diagnosis (N (%))	*No*	21 (36.21)	13 (61.90)	0.075^c^	–	–	–
	*Yes*	37 (63.79)	8 (38.10)				
Other substance use disorders (N (%))	*No*	–	–	–	8 (17.78)	13 (39.24)	0.075^c^
	*Yes*				37 (82.22)	21(61.76)	

**Notes.**

Abbreviations: BMI body mass index

a *p*-value from Student’s-test.

b *p*-value from Wilcoxon-test.

c *p*-value from Chi-square-test.

In this study, we used the Kolmogorov–Smirnov test with Lilliefors correction to analyze normality of data. We used Levene’s test to analyze homoscedasticity of the data.

All statistical analyses were performed using R-commander version 3.3.2 free software and GraphPad Prism version 5.04 software (GraphPad Software, San Diego, CA, USA). A *p*-value <0.05 was considered statistically significant. The specific statistical analysis used is indicated in the text and in each figure caption.

## Results

### Socio-demographic characteristics

The description of the socio-demographic variables of the participants is presented in Table 1. A total of 160 subjects met the eligibility criteria for this study and were divided into the cocaine (*N* = 79) and control (*N* = 81) groups.

The average age of the participants in the cocaine abstinent group was 35 years old, and the average BMI was 26. We found significant differences between patients with CUD and the controls in the marital status variable (*p* < 0.05). Cocaine patients had a significantly lower educational level and a higher percentage of psychiatric treatments compared with the control group—(*p* < 0.001).

A total of 57% of cocaine patients were diagnosed with dual diagnosis, which includes presenting with at least one psychiatric disorder throughout life (i.e., mood, anxiety, psychotic or personality disorders (antisocial and borderline)). In addition, 73% of cocaine patients were diagnosed with comorbid substance use disorders (e.g., alcohol, heroin, cannabis, benzodiazepines, hallucinogens or other stimulants). The length of abstinence from cocaine in these patients was 133 days, and the mean cocaine symptom severity was 8 CUD-criteria at the time of recruitment.

### Plasma levels of inflammatory signaling proteins in subjects with CUD and in controls

As shown in Fig. 1, the estimated marginal means of eotaxin-1, IFNγ, IL-4, IL-8, IL-17α, MIP-1α and TGFα in the plasma are presented according to the history of cocaine use (the cocaine and control groups). The plasma concentrations of these factors were analyzed by ANCOVA using ‘cocaine use’ as the main factor and controlling for age, sex and BMI.

We found a significant main effect of ‘cocaine use’ on plasma levels of IL-17α –(*F*_1,133_ = 20.713, *p* < 0.001), MIP-1α –(*F*_1,147_ = 26.444, *p* < 0.001) and TGFα –(*F*_1,540_ = 5.251, *p* < 0.05). Thus, cocaine patients showed lower levels of these inflammatory proteins than the control group. In contrast, we found no differences between both groups in the levels of eotaxin-1, IFNγ, IL-4 or IL-8.

### Impact of psychiatric comorbidity on plasma levels of inflammatory signaling proteins in subjects with CUD

Cocaine patients were divided into subgroups according to the diagnosis of psychiatric comorbidity. Additionally, we used two different criteria of classification to explore the effects of psychiatric comorbidity on these inflammatory factors: (a) Diagnosis of comorbid substance use disorders and (b) Dual diagnosis with other mental health disorder(s). A description of these patients is shown in Table 2.

A first classification of the cocaine group was performed according to the diagnosis of other substance use disorders (yes (*N* = 58); no (*N* = 21)). The comparison between both subgroups showed that there were no significant differences in age, BMI, sex, psychiatric treatment, age of cocaine initiation or length of abstinence. However, we found significant differences in the cocaine symptom severity –(*p* < 0.001), and patients with comorbid substance use disorders had higher cocaine severity than patients with no other substance use disorders (8.4 vs. 7.0 criteria). Although patients with substance use disorders had an elevated prevalence of dual diagnosis (64%) compared with patients with no other substance use disorders (38%); however, this difference did not reach statistical significance (*p* = 0.075).

An additional classification was performed according to the detection of dual diagnosis (yes (*N* = 45); no (*N* = 34)). We found no differences in BMI, sex, psychiatric treatment, age of cocaine initiation or length of abstinence. In this case, we observed significant differences in age (*p* < 0.05) and in the cocaine symptom severity (*p* < 0.001). Further, patients with dual diagnosis were older (36.5 vs. 32.7 years) and had a more severe form of CUD (9.2 vs. 6.5 criteria) than patients with no dual diagnosis. Furthermore, these patients with dual diagnosis had an increased prevalence of comorbid substance use disorders (82%) relative to patients with no dual diagnosis (62%), but this difference did not quite reach statistical significance (*p* = 0.075).

### Impact of dual diagnosis on plasma levels of inflammatory signaling proteins in subjects with CUD

Figure 2 shows the estimated marginal means of eotaxin-1, IFNγ, IL-4, IL-8, IL-17α, MIP-1α and TGFα in the plasma of cocaine patients, who were grouped according to the presence of dual diagnosis. The plasma concentrations of these inflammatory markers were analyzed by ANCOVA using ‘dual diagnosis’ as the main factor and controlling for age, sex and BMI.

**Table 3:** Cocaine symptom severity on plasma levels of inflammatory signaling proteins.

Variable	Concentration (pg/mL) (mean (SD))						
	Eotaxin-1	IFNγ	IL-4	IL-8	IL-17α	MIP-1α	TGFα
Mild/moderate (0–8)criteria^a^ ***N* = 38**	35.16 (1.09)	2.13 (1.26)	1.38 (1.37)	4.02 (1.08)	0.27 (1.21)	1.88 (1.19)	1.71 (1.46)
**Severe** (9–11)criteria^a^ ***N* = 41**	38.46 (1.09)	2.47 (1.25)	1.45 (1.41)	4.04 (1.08)	0.35 (1.21)	1.96 (1.19)	0.95 (1.70)
*p* value^b^	0.405	0.604	0.908	0.949	0.280	0.837	0.315

**Notes.**

a Based on DSM-IV-TR abuse and dependence criteria.

b *P*-value from ANCOVA analysis. It denotes significant differences when it is <0.05.

There was a significant main effect of ‘dual diagnosis’ on the plasma levels of TGFα –(*F*_1,190_ = 6.812, *p* < 0.05). Thus, cocaine patients with dual diagnosis showed lower TGFα levels than patients with no dual diagnosis. Consequently, the presence of dual diagnosis in the cocaine group enhanced a decrease in TGFα levels relative to the levels in the control group.

### Impact of cocaine symptom severity on plasma levels of inflammatory signaling proteins in subjects with CUD

Because we observed significant differences in the cocaine symptom severity when cocaine patients were grouped according to psychiatric comorbidity (comorbid substance use disorders and dual diagnosis), we explored the impact of the degree of cocaine severity on these circulating inflammatory proteins. Therefore, the cocaine group was divided into 2 subgroups as follows: mild/moderate ((0–8 criteria) *N* = 38) and severe ((9–11 criteria) *N* = 41) CUD.

Similar to previous analyses, plasma concentrations of these proteins were analyzed by ANCOVA using ‘cocaine severity’ as the main factor and controlling for age, sex and BMI. However, as shown in Table 3, we found no differences between both subgroups.

## Discussion

The search for potential biomarkers might enable a better stratification of patients to establish therapeutic subgroups. These biomarkers might arise from the activity of biological systems involved in the pathogenesis of mental disorders. There is evidence that the immune system plays an important role in the pathogenesis of mental disorders and might be a source for diagnostic biomarkers relevant for the treatment of addictive disorders and associated psychiatric diseases (Fox et al., 2012; Araos et al., 2015; García-Marchena et al., 2017a; García-Marchena et al., 2017b). In the present study, we attempted to establish potential biomarkers of cocaine consumption and/or comorbid mental disorders by identifying certain, cytokines, chemokines and growth factors in the plasma of patients with these disorders. Previous studies (Araos et al., 2015; Pedraz et al., 2015) have revealed that the circulatory/immune system-related signal or growth factors are associated with CUD. The present results extend these observations and indicate that IL-17α, MIP-1α and TGFα are altered in subjects diagnosed with CUD during abstinence, although there was no association with the cocaine symptom severity, and that TGFα levels are also influenced by the presence of dual diagnosis. Although certain studies revealed positive associations between cytokines, chemokines, circulating growth factors and CUD (Sáez et al., 2011; Levandowski et al., 2016; Scherer et al., 2016), other studies reported a decreased expression of inflammatory proteins in CUD patients.

For instance, cocaine abusers were found to have decreased IL-10 levels compared with the levels found in social drinkers (Fox et al., 2012), and cocaine-dependent volunteers showed a decrease in the expression of cytokines such as TNFα and IL-6 (Irwin et al., 2007). Therefore, the role of these inflammatory signals in the neurobiology of addiction is not yet fully understood.

Cytokines and other inflammatory mediators are produced by several types of cells, mainly immune cells (e.g., lymphocytes and macrophages), and act through receptors in the regulation of crucial processes such as inflammation and embryogenesis. Some of these signals can cross the blood brain barrier and produce an inflammatory state that has been linked to dysfunctions on ascending monoaminergic systems (Petrulli et al., 2017). In fact, cytokines and their receptors are expressed in microglia, which are immune cells present in the brain that participate in processes such as remodeling and synaptic pruning (Paolicelli et al., 2011), as well as in astrocytes and neurons.

The decreased plasma levels of IL-17α, MIP-1α and TGFα are similar to that described in the same population for other cytokines and chemokines such as TNFα, MCP-1 and SDF-1 (Araos et al., 2015). Although cocaine has been described to produce a pro-inflammatory state, the decrease in the levels of IL-17α, MIP-1α and TGFα can be interpreted as a compensatory effect that reduces the cocaine-induced inflammatory tone along the period of abstinence in comparison with control subjects. A similar phenomenon has been described for certain growth factor in our outpatient cohort of cocaine patients (Pedraz et al., 2015). However, none of the proteins that were analyzed in the present study were associated with CUD severity, unlike our previously described findings with IL-1β, fractalkine or SDF-1 as severity-sensitive factors (Araos et al., 2015).

As currently described in the literature, IL-17α plays a key role in autoimmune diseases in the CNS, such as multiple sclerosis (Zimmermann et al., 2017). However, there are no studies showing that IL-17α is involved in the effects of cocaine or other abused drugs. Regarding MIP-1α, this chemokine contributes to the modulation of immune responses, and MIP-1α is believed to be important in the pathogenesis of autoimmune and infectious diseases, as well as cancer (Snyder-Cappione et al., 2010). Recently, we have described that concentrations of MIP-1α are mildly affected in patients with alcohol use disorders (García-Marchena et al., 2017a). Although there is no scientific evidence linking this chemokine to the neuropharmacology of cocaine, it has been described that cocaine use activates the release of MIP-1α and promotes opening of the blood brain barrier, facilitating not only neuroinflammation but also viral infection from drug injectors (Zhang et al., 1998). Considering that neurobiological changes associated with peripheral inflammatory states facilitate the action of psychostimulants (Petrulli et al., 2017), this clear association between MIP-1α and cocaine use demands further neurobiological studies.

It has been discovered that TGFα is related to a number of diseases. This ligand of the epidermal growth factor receptor has been proposed as a prognostic biomarker for gastric carcinoma (Fanelli et al., 2012), also for melanoma (Tarhini et al., 2014). Concerning CNS, TGFα is related to neurogenesis (Cooper & Isacson, 2004) and the decrease of TGFα in the plasma of cocaine patients might be related to a decrease in neurogenesis, which has been reported after acute administration of cocaine in animal models (Blanco-Calvo et al., 2014). This inhibition of neurogenesis might be associated with persistent rewarding memories for cocaine, and therefore, a decreased expression of TGFα might facilitate the persistence of cocaine abuse (Deschaux et al., 2014). Additionally, genetic models lacking TGFα in the brain result in super sensitivity to the psychostimulant effect of cocaine in a manner similar to that described for inflammation (Stanwood & Levitt, 2007). However, although a study in a population with heroin abuse disorders shows elevated levels of TGFα in injecting drug users with active consumption (Piepenbrink et al., 2016), to our knowledge, there are no studies relating TGFα to CUD.

Over the last decade, a growing number of studies have explored the potential role of cytokines, chemokines and growth factors in populations with mental disorders (Raison, Capuron & Miller, 2006; Ogłodek et al., 2015; Van Varsseveld et al., 2015; Engler et al., 2017; Michopoulos et al., 2017; Notter et al., 2017) and/or drug addiction, including CUD (Irwin et al., 2007; Parikh et al., 2014; Levandowski et al., 2016). Thus, certain studies have found reduced circulating cytokines in patients with generalized anxiety before and after Mindfulness treatment, when compared to their controls (Hoge et al., in press). Plasma levels of IL-17 and IL-23 are altered in schizophrenic patients relative to the levels in their controls (Li et al., 2016). Additionally, longitudinal studies described how personality traits predict IL-6 levels (Turiano et al., 2013).

An increase in IL-1β levels has been observed in patients with CUD with dual diagnosis compared to patients with CUD without a dual diagnosis (Araos et al., 2015). We believe that, together with monitoring cytokines, chemokines and growth factors, it is also necessary to characterize these patients with and without dual diagnosis, since they are patients with different therapeutic needs and approaches (Balhara, Kuppili & Gupta, 2017).

Related to the above, TGFα emerges as the only signal associated with both CUD and dual diagnosis. To our knowledge, there is no scientific literature on the relationship between CUD, dual diagnosis and plasma levels of TGFα.

In any case, the evaluation and validation of TGFα as a suitable candidate for a potential biomarker of both CUD and dual diagnosis could open new research lines for understanding the complexities of cocaine addiction and the associated dual diagnosis.

The evaluation of cytokines, chemokines and growth factors in plasma could improve the stratification of CUD patients undergoing treatment and complement therapeutic interventions, including the high risk of dual diagnosis. Additional studies would be needed to examine new molecules of the immune system in order to elucidate their role in the etiology of CUD.

Several limitations should be considered when discussing the present findings. First, larger studies are needed because variability is common when considering the association of immune signals with mental disorders. As an example, a meta-analysis of depressive patients with matched controls and their relationship with circulating levels of plasma cytokines shows that there is a considerable heterogeneity of results (Köhler et al., 2017). Second, it would also be necessary to include samples of depressed or anxious patients with no diagnosis of substance use disorders throughout life in order to compare CUD populations with or without dual diagnosis. Third, the small number of female patients is an important limitation of the present study. It would be necessary to increase the sample population of women in order to have a more comprehensive view of the results obtained. Fourth, it would be interesting to include in future studies subjects with active cocaine consumption to observe the effects of the presence of cocaine at circulating levels. It would also be necessary to control the pathway of confounding pharmacological treatment; as such, controlling the medical use of anti-depressants, anxiolytics and anti-psychotics would give more consistency to the current findings.

Finally, there is a need for integrating all the information concerning this multiplicity of inflammatory signals in a single model of cocaine addiction. Although certain factors such as TGFα might contribute to important aspects of cocaine addiction and associated psychiatric comorbidities, the complexity of the interactions of these signaling inflammatory proteins falls beyond our current understanding. Further basic and clinical research is needed to elucidate the role of TGFα and other factors in the pathogenesis of addiction and their utility as potential clinical biomarkers.

We conclude from this study that TGFα could be a potential biomarker of CUD and dual diagnosis in abstinent patients; moreover, additional studies are needed to investigate and validate the effects of TGFα in patients with CUD.

##  Supplemental Information

10.7717/peerj.3926/supp-1 Supplemental Information 1 Sociodemographic psychiatric and molecular levels

## Additional Information and Declarations

### Data Availability

The following information was supplied regarding data availability: The raw data has been provided as a Supplemental File.
